# Acceptability and face validity of two mental health screening tools for use in the routine surgical setting

**DOI:** 10.1186/s40359-021-00672-w

**Published:** 2021-10-30

**Authors:** Kate E. McBride, Daniel Steffens, Tim Lambert, Nick Glozier, Rachael Roberts, Michael J. Solomon

**Affiliations:** 1 RPA Institute of Academic Surgery (IAS), Royal Prince Alfred Hospital and University of Sydney, PO Box M157, Sydney, NSW Australia; 2 Sydney Medical School, Faculty of Medicine and Health, University of Sydney, Sydney, NSW Australia; 3 Surgical Outcomes Research Centre (SOuRCe), Sydney, NSW Australia; 4 ccCHiP, Charles Perkins Centre, University of Sydney, Sydney, NSW Australia; 5 Brain and Mind Centre, University of Sydney, Sydney, NSW Australia

**Keywords:** Mental illness, Surgery, Preoperative screening, Acceptability, Face validity

## Abstract

**Background:**

Preoperative assessment of mental health rarely occurs within routine surgery. Any screening tool selected to form part of this process must be deemed practical, acceptable and valid by clinicians and consumers alike. This study aims to assess the acceptability and face validity of two existing mental health screening tools to select one for further development and use in the routine surgical setting.

**Methods:**

A survey of clinicians and consumers was conducted from October 2020 to March 2021 at a tertiary hospital in Sydney, Australia. Using a Likert scale (1–5, lowest to highest rating), the clinicians evaluated four domains for acceptability and two for validity (six overall) and the consumers four domains for acceptability and one for validity (five overall) on the preoperative use of the amended *Kessler Psychological Distress Scale (K10)* and the *Somatic and Psychological Health Report-12 (SPHERE-12)*. Consensus was achieved through a rating of 4 or 5 being given by 70% or more of participants with domains able to remain unchanged. Free text responses were analysed into themes.

**Results:**

A total of 73 participants (51 clinicians; 22 consumers) were included. The K10 received consensus scores (≥ 70%) in four out of six domains for clinicians (4/4 acceptability; 0/2 validity), and all five domains for consumers (4/4 acceptability; 1/1 validity). The SPHERE-12 received consensus scores (≥ 70%) in three domains for clinicians (3/4 acceptability; 0/2 validity), and three domains for consumers (3/4 acceptability; 0/1 validity). Six qualitative themes were described including (1) amendments to tool structure and language; (2) scale response options; (3) difficulty with somatic questions; (4) practicality and familiarity with K10; (5) challenges for specific patient cohorts and (6) timing considerations for patients.

**Conclusion:**

Adequate acceptability was established for the K10. However further development is required to strengthen its validity for this specific surgical cohort and purpose. Future research to determine the feasibility and acceptability of implementing and using the K10 in the routine surgical setting is now needed.

**Supplementary Information:**

The online version contains supplementary material available at 10.1186/s40359-021-00672-w.

## Introduction

In order to provide high-value care within surgery, identifying patient-specific preoperative risk factors is essential for optimising surgical treatment. Whilst screening for medical comorbidities such as cardiovascular or respiratory conditions is well embedded and occurs routinely in preparing patients for surgery, consideration of mental health is rarely, if ever, undertaken [1]. This is concerning, given the significantly poorer surgical outcomes experienced by patients with serious mental illness (SMI), compared to the general population. This includes increased rate of postoperative complications, length of stay in hospital and hospital readmissions across a range of procedures and health systems internationally [2–5]. SMI, which is defined as a mental, behavioural and/or emotional disorder that has episodic, recurrent or persistent features resulting in severe impairment [6], impacts on three percent of the population and includes a highly vulnerable group of patients due to the range of health, social and occupational challenges they face [7].

Given this known gap in surgical practise and the critical needs of these patients, it is apparent that simple mental health screening may be a beneficial mechanism for proactively identifying those patients who need further support with their mental health. Although limited, there is evidence to show that even modest preoperative psychological interventions can have favourable effects on surgical patient outcomes by matching a range of interventions to the needs of the patients [8]. In addition, and perhaps most critically, patients with SMI have described their support and desire for having the status of their mental health acknowledged prior to surgery and identified screening as a potential solution [9]. Currently there are no known mental health screening tools designed specifically for use in the routine surgical setting that cover a broad range of psychological conditions and symptoms. It is also imperative that any existing tool selected and amended for this purpose, is able to be seamlessly included into the existing preoperative planning processes and deemed practical, acceptable and valid to both clinicians and consumers. As such, the aim of this study is to assess the acceptability and face validity of two existing mental health screening tools using clinician and consumer feedback, to enable selection of one for further development and use in the routine surgical setting.

## Methods

### Study design and ethics

A survey of clinicians and consumers was conducted on Royal Prince Alfred (RPA) Hospital campus in Sydney, Australia between October 2020 and March 2021 to measure the acceptability and face validity of two amended mental health screening tools with the intention to select one for further development and pilot testing. A focus on colorectal and cardiothoracic surgical specialties for both the clinicians and consumers was undertaken based on the higher rates of surgical patients with mental health comorbidities identified in these groups [2]. Ethics approval was granted by the Sydney Local Health District Ethics Review Committee—Royal Prince Alfred (RPA) Hospital Zone (X20-0386).

### Study sample

Feedback was sought from a minimum of 50 clinicians and 20 consumers to voluntarily participate from the following groups:

(i) *Clinicians:* Consultant level clinicians potentially involved in the surgical care of people with SMI including cardiothoracic and colorectal surgeons, anaesthetists, psychiatrists, psychologists and mental health or surgical clinical nurse consultants (CNC) were invited to participate in the study via email, which included a link to the online survey and a softcopy, by their own head of department (at arm’s length from the study investigators);
(ii) *Consumers:* Both surgical and mental health consumers were invited to participate in the study. Postoperative adult inpatients from either the cardiothoracic or colorectal wards at RPA Hospital were provided with a hardcopy survey and advised to return it to the Nursing Unit Manager. People with self-reported mental illness who were members of the consumer group; *SLHD Lived Experiences Advisory Panel (LEAP)* meeting and/or attending the *Collaborative Centre for Cardiometabolic Health in Psychosis (ccCHIP)* outpatient clinic were either emailed the survey with a link to the online survey or provided with a hardcopy survey by their nursing team.

Recruitment into the study closed once the minimum number of participants had responded. Due to the ethical requirement for the study investigators to stay at arms length from the participants, the overall response rate was unable to be determined and minimum response numbers were sought instead.

### Amended mental health screening tools

Following a comprehensive literature review of 470 studies completed by these authors (paper in submission under review), two tools were identified from a detailed review of 32 tools as being potentially suitable for use in the routine surgical setting; the *Kessler Psychological Distress Scale* (K10) [10], which includes 10-items measuring anxiety, depression and distress symptoms over the last 30 days using a 5-point Likert scale (1–5) and the *Somatic and Psychological Health Report-12 (SPHERE-12),* which includes 12-items measuring anxiety, depression, fatigue and somatization captured within two sub-scales (PSYCH and SOMA) over the last few weeks using a 3-point Likert scale (1–2) [11]. Their features included being self-report, applicable to adults with a broad range of psychological disorders and symptoms, brief to complete, easy to score, free to use and with adequate psychometric properties across internal consistency, test re-test reliability, validity and responsiveness. To ensure the tools were fit-for-purpose for use in the preoperative environment and focusing on people with SMI, the investigators, with expertise in both psychiatry and surgery, developed four additional questions to add at the end of the tools, which were initially derived from several sources [12, 13], and then refined following consultation with colleagues and a consensus meeting.The four additional questions were added under the heading ‘Overview of your mental health and wellbeing’ and included the following: (1) Do you currently see or have you ever seen anyone for assistance with your mental wellbeing?, (2) Have you ever been told by a healthcare worker that you have one of the following conditions?, (3) Do you, or your carer/support person, have any serious concerns about your upcoming surgical procedure affecting your mental health and wellbeing? e.g. about your medication, your treatment, how you feel etc., (4) Would you, or your carer/support person, like to talk with someone about your mental health before your surgical procedure or receive information about mental health services available for you to use? (Additional file 1).

### Acceptability assessment

Following review of the K10 and SPHERE-12, both the clinicians and consumers anonymously completed four survey questions exploring the following domains on each tool as a whole; (1) clarity of wording, (2) suitability of the tool length, (3) ease of use, and (4) their perception on the willingness of patients to complete the tool. Both participants groups used a five-point Likert scale to rate each domain (score range 1–5 with 1 = very unclear/unsuitable/not easy to use/unwilling to complete and 5 = very clear/suitable/easy to use/willing to complete).

In addition, to further measure acceptability, the readability and comprehension difficulty of the K10, SPHERE-12 and the additional four questions added were assessed using the commonly used ‘Flesch Reading Ease’ and ‘Flesch-Kincaid grade level’ methods [14–16]. These measures use word and sentence length to provide the readability and education level of a text. Flesch readability scores range from 0 to 100 with scores ≥ 60 indicating a document is well written and easy to follow. A reading grade aimed at 6th–7th grade (11–12 years old) has been shown to be suitable for patient level information, which is indicated by corresponding scores between 6 and 7 [14–16]. Microsoft Word was used to provide these reading statistics, which has been shown to be valid and reliable [17].

### Face validity assessment

After reviewing the K10 and SPHERE-12, the clinicians anonymously completed two survey questions exploring the (5) usefulness and (6) thoroughness of each tool as a whole. The surgical and mental health consumers rated one domain exploring the (5) design of the tool. Again both participants groups used a five-point Likert scale to score each question (score range 1–5 with 1 = not useful/unthorough/not well designed and 5 = very useful/thorough/well designed), which was developed using other measures of face validity as a guide for this assessment [18, 19]. Participants were advised ‘Face Validity’ is assessing whether the tools are appropriate for the aim of preoperative mental health screening for all routine surgical patients.

Overall the clinicians answered six and the consumers’ five survey questions in total, for each tool (Table 1). There was also a free text option for all participants to make comments or suggested changes to each of the tools.

**Table 1:** Survey questions for clinicians and consumers

*Clinicians*	
(1) Clarity of wording	How clearly do you think this tool is worded for patients to complete before their surgery?
(2) Suitability of length	How suitable is the length of this tool for patients to complete before their surgery?
(3) Ease of use	How easy is this tool to use for patients to complete before their surgery?
(4) Thoroughness	How thoroughly does this tool screen the mental health of patients before their surgery?
(5) Usefulness	How useful is this tool for preoperatively screening the mental health of patients before their surgery?
(6) Willingness to use	How willing do you think future surgical patients will be to complete this tool?
*Consumers*	
(1) Clarity of wording	How clearly do you think this tool is worded? e.g. use of simple and clear language
(2) Design	How well do you think this tool is designed? e.g. the presentation, layout, and flow of questions
(3) Suitability of length	How suitable is the length of this tool to complete? e.g. number and type of questions and answers
(4) Ease of use	How easy do you think this tool is to use? e.g. is it simple
(5) Willingness to use	How willing do you think future surgical patients will be to complete this tool?

### Data analysis

Survey response data was stored within a Research Electronic Data Capture (REDCap) database. A basic descriptive analysis was performed according to participant grouping of clinicians and consumers using SPSS Software version 25 (IBM Corporation, Armonk, NY, USA).Consensus was achieved through a rating of 4 or 5 being given by 70% or more of participants, with domains able to remain unchanged.This was based on similar approaches previously described whereby between 60 and 80% of support is required [18, 20, 21]. Questions that received lower consensus percentages were established as areas requiring future revision to improve the tool for use in the routine surgical setting.. The Shapiro–Wilk test was used in order to determine normality. The Chi-square test or the Fisher exact test was used to determine the difference between clinicians (i.e. Anaethetists, Clinical Nurse Consultants, Psychiatrists, Psychologists, and Surgeons) and consumer groups (i.e. mental health and surgical consumers) who rated the mental health screening tools a low (< 4) or high score (≥ 4) across the acceptability and face validity domains assessed *P* < 0.05 was considered statistically significant for all the analyses.

The free text was quantitatively described and qualitatively analysed into themes through thematic analysis as described by Braun and Clarke [22]. This flexible method was selected given the views of the participants were not known and it is an under-researched area. Responses were manually coded into themes and independently assigned by two investigators (KM and DS), who took a realist or straightforward interpretation of the data. Once the codes were determined, the investigators worked together to sort the codes into main themes for both the clinician and consumer groups. Both the quantitative and qualitative data was then synthesised by the investigators to select one tool for further development and future pilot testing.

## Results

A total of 73 participants provided feedback on the proposed use of the amended K10 and SPHERE-12 screening tools in the routine surgical setting. This included 51 clinicians, comprising seven colorectal and three cardiothoracic surgeons, 10 anaesthetists, 10 psychiatrists, 10 psychologists, and five colorectal, four mental health, one cardiothoracic and one preadmission CNC. Of the 22 consumer participants, 13 were surgical consumers and nine were mental health consumers.

For the clinicians, consensus was achieved for all four acceptability domains of the K10 to remain unchanged. The SPHERE-12 received consesus in three out of the four acceptability domains. Neither the K10 or the SPHERE-12 achieved consensus in either of the two face validity domains (Table 2).There was a significant difference found between the consensus percentages reported across the disciplines for both validity domains of usefulness and thoroughness of the K10 and for the domains of length, usefulness and the willingness to complete for the SPHERE-12.

**Table 2:** Percentage of clinician who rated the mental health screening tools a score of 4 or 5

Clinician rating	Acceptability				Face validity	
	Clarity	Length	Ease of use	Willingness to complete	Usefulness	Thoroughness
*Kessler psychological distress scale (K10)*						
Anaesthetists (*n* = 10)	8 (80%)	8 (80%)	8 (80%)	6 (60%)	5 (50%)	6 (60%)
Clinical nurse consultants (*n* = 11)	10 (91%)	11 (100%)	10 (91%)	8 (80%)^a^	9 (90%)^a^	8 (80%)^a^
Psychiatrists (*n* = 10)	10 (100%)	8 80%)	8 (80%)	5 (50%)	1 (10%)	3 (30%)
Psychologists (*n* = 10)	6 (60%)	7 (70%)	8 (80%)	7 (70%)	7 (70%)	1 (10%)
Surgeons (*n* = 10)	9 (90%)	8 (80%)	8 (80%)	9 (90%)	8 (80%)	8 (80%)
*P* value	0.132	0.475	0.951	0.313	0.002	0.004
Overall (*n* = 51)	43 (84%)	42 (82%)	42 (82%)	35 (70%)^a^	30 (60%)^a^	26 (52%)^a^
*Somatic and psychological health report-12 (SPHERE-12)*						
Anaesthetists (*n* = 10)	9 (90%)	10 (100%)	7 (70%)	7 (70%)	5 (50%)	5 (50%)
Clinical Nurse Consultants (*n* = 11)	8 (73%)	6 (55%)	7 (64%)	3 (30%)^a^	3 (30%)^a^	3 (30%)^a^
Psychiatrists (*n* = 10)	8 (80%)	9 (90%)	9 (90%)	1 (10%)	0 (0%)	2 (20%)
Psychologists (*n* = 10)	9 (90%)	9 (90%)	7 (70%)	8 (80%)	6 (60%)	7 (70%)
Surgeons (*n* = 10)	10 (100%)	10 (100%)	9 (90%)	9 (90%)	8 (80%)	6 (60%)
*P* value	0.425	0.012	0.477	0.001	0.005	0.140
Overall (*n* = 51)	44 (86%)	44 (86%)	39 (77%)	28 (56%)^a^	22 (44%)^a^	23 (46%)^a^

Based on 5-point Likert Scale (range 1–5) with 5 indicating highest rating of consensus; Data presented as frequency (percentage) of score ≥ 4; *P* < 0.05 indicate statistical significancy

^a^Indicate missing data (N = 1)

For the consumers, consensus was achieved for all five domains of the K10 and three out of five for the SPHERE-12(Table 3). There was a significant difference between the consensus percentages reported for the domain of clarity for the SPHERE-12, with the mental health consumers rating it higher than the surgical consumers.

**Table 3:** Percentage of consumers who rated the mental health screening tools a score of 4 or 5

Consumer rating	Acceptability			Face validity	
	Clarity	Length	Ease of use	Willingness to complete	design
*Kessler psychological distress scale (k10)*					
Mental health (*n* = 9)	8 (89%)	7 (78%)	7 (78%)	7 (78%)	7 (78%)
Surgical (*n* = 14)	13 (93%)	12 (86%)	13 (93%)	12 (86%)	11 (79%)
*P* value	0.742	0.624	0.295	0.624	0.964
Overall (*n* = 23)	21 (91%)	19 (83%)	20 (87%)	19 (83%)	18 (78%)
*Somatic and psychological health report-12 (SPHERE-12)*					
Mental health (*n* = 9)	9 (100%)	7 (78%)	8 (89%)	7 (78%)	8 (89%)
Surgical (*n* = 14)	9 (64%)	11 (92%)^a^	10 (91%)^b^	8 (62%)^c^	7 (50%)
*P* value	0.043	0.368	0.881	0.421	0.056
Overall (*n* = 23)	18 (78%)	18 (86%)^a^	18 (90%)^b^	15 (68%)^c^	15 (65%)

Based on 5-point Likert Scale (range 1–5) with 5 indicating highest rating of consensus; Data presented as frequency (percentage) of score ≥ 4; *P* < 0.05 indicate statistical significancy

^a^Indicate missing data (N = 2)

^b^Indicate missing data (N = 3)

^c^Indicate missing data (N = 1)

The free text option was utilised by 33 (64.7%) of clinicians and 15 (68.2%) of the consumers. For the K10, supportive comments to use the tool were provided by 9 (27.3%) clinicians and 10 (66.7%) consumers. Unsupportive comments were provided by 5 (15.2%) clinicians and 0 (0%) consumers. For the SPHERE-12, supportive comments to use the tool were provided by 5 (15.2%) clinicians and 7 (46.7%) consumers. Unsupportive comments were provided by 14 (42.4%) clinicians and 3 (20.0%) consumers.

The feedback was qualitatively analysed into the following six themes, which are summarised in Table 4; (1) Amendments to tool structure and language, (2) Scale response options (3 vs. 5), (3) Difficulty with inclusion of somatic symptoms in SPHERE-12, (4) Greater practicality and familiarity with using the K10, (5) Challenges for specific patient cohorts to complete the tool, and (6) Pre-operative timing consideration for surgical patients to complete the tool.

**Table 4:** Qualitative themes and example quotes from participants on the mental health screening tools

Clinicians (n = 33 respondents)	Response # (%)	Consumers (n = 15 respondents)	Response # (%)
*(1) Amendments to tool structure and language*			
[3]—Ask if they are interested in information about MH referrals. Ask about drug and alcohol issues which are also very common	28 (84.5%)	[57]—I think that the last part of question 11 could be laid out better	3 (20.0%)
[27]—I would reorder the options for question 11 and start with anxiety or depression first rather than Schizophrenia. Q.13—Have 'other' option		[94]—Also interested about non-inclusion of carer question – e.g. do you have or would you like a support person or carer?	
*(2) Scale response options (3 vs. 5)*			
[49]—I think the 5 point scale is more likely to have more sensitivity in picking up problems. The second scale (SPHERE-12) is also liable to be misinterpreted more easily by a patient with a serious mental illness, is less clear and direct and appears more casual	12 (36.4%)	[57]—I found the wording and the categories in questions 1–12 somewhat difficult in SPHERE e.g. choosing between answers a b and c present problems in understanding. I think that never, sometimes or always would be easier choices	7 (46.7%)
[68]—Potential for patients to get confused / overwhelmed with 5 options, especially as filling out many forms in the preadmission clinic and requires increased mental load / capacity		[74]—I feel like the 3-point scale will cause people to err on the low side. Maybe that it ok, but perhaps a 4 point scale may be more accurate	
*(3) Difficulty with inclusion of somatic symptoms in SPHERE-12*			
[82]—Depending on surgical procedure, the SPHERE-12 is likely to increase scores and reduce specificity particularly with the somatic questions	9 (27.3%)		
[85]—Somatic symptom questions not specific to mental health in a pre-surgical setting is a problem			
*(4) Greater practicality and familiarity with using the K10 tool*			
[6]—In broad terms the SPHERE while locally developed is relatively infrequently used	8 (24.2%)		
[48]—As K10 is standard form for MH and has eMR version, more likely than SPHERE-12 to have comparative measures			
*(5) Challenges for specific patient cohorts to complete a tool*			
[31]—May be difficult to use for thought disordered patients and/or paranoid patients	5 (15.2%)		
[45]—There will be the obvious difficulty with NESB patients			
*(6) Preoperative timing consideration for surgical patients to complete the tool*			
		[14]—I think surgical patients may be too geared up by an operation to answer the survey adequately	2 (13.3%)
		[80]—Patients would in general be willing to fill in survey but not in first week	

The ‘Flesch Reading Ease’ measure was 78.4 for the K10 indicating it was the simplest and easiest to read and well above the minimum scores required. The SPHERE-12 and the four additional questions added to the tools scored lower at 59.7 and 59.2 respectively, but these were still within the range considered well written and easy to follow (≥ 60). The ‘Flesch-Kincaid grade level’ measure was 5.4 for the K10 indicating the lowest reading grade level at the 5th–6th grade, 6.9 for the SPHERE-12 equivalent to the 6th–7th grade and 8.9 or equivalent to the 8th–9th grade for the four additional questions added to the tools.

## Discussion

Feedback from both clinicians and consumers was used to measure the acceptability and face validity of two amended mental health screening tools, the K10 and SPHERE-12, for potential use in the routine surgical setting. Overall the consensus achieved on the domains of the K10 was higher than the SPHERE-12 for both clinicians and consumers. The qualitative feedback was also more supportive of using the K10. This included greater practicality and familiarity with the K10 due to it being widely used in mental health services across Australia, the larger number of response options perceived as being more comprehensive for both patients and clinicians, and the preferred style of questioning. Furthermore, the readability and comprehension difficulty of the K10 was also found to be most suitable using the ‘Flesch Reading Ease’ and ‘Flesch-Kincaid grade level’ measures. Based on this collective quantitative and qualitative feedback, the K10 tool is recommended for further development, implementation and evaluation in the routine surgical setting.

It is important to note the findings presented in this study form the first phase of a larger project, which will involve pilot testing the feasibility, acceptability and accuracy of implementing the selected K10 tool into the preoperative screening process for all routine surgical patients booked for cardiothoracic and colorectal surgery. These specialties were chosen based on the results of a previous study demonstrating the higher prevalence of SMI in these groups [2]. This pilot study will also determine the feasibility and acceptability of providing additional mental health support to those surgical patients identified by the K10 tool. It will also provide critical data on the capacity of the tool to identify patients with SMI, which is known to be a considerable challenge [23, 24]. The information gathered from the pilot will then assist in the future planning of a much-needed randomised control trial to determine whether proactive management of patient’s mental health through preoperative screening can improve the surgical outcomes and experience for patients with SMI.

Prior to the commencement of the pilot and based on the results of this study, it is evident the K10 may benefit from further development to strengthen its face validity to be used in the routine surgical setting. This includes improving the ‘usefulness’ and ‘thoroughness’ of the tool. Interestingly three main themes from the qualitative feedback emerged that related specifically to these two domains. Firstly, the number of response options on the tool was highly polarizing amongst all participant groups. On the one hand, the five response options of the K10 was framed as being more thorough in providing patients with additional options to describe their mental health and more useful in giving clinicians greater detail about the patient. Conversely, the three response options of the SPHERE-12 was felt by both clinicians and patients to be simpler, less overwhelming and more useful for patients to articulate their symptoms. Secondly, the inclusion of somatic questions on the tools was highlighted to be problematic for surgical patients, whereby it might result in false positives, and reduce the usefulness of the screening tools for this particular cohort. Clinicians reported this as a particular concern with the use of the SPHERE-12, which includes six questions focused on somatic symptoms. Finally, the thoroughness of the tools in identifying patients with SMI was raised with conflicting feedback. Overall clinicians viewed the four additional questions as being a positive inclusion that added value and strength to the tools. In contrast, several psychiatrists indicated that due to the known mistrust of the health system and fear of stigmatization, patients with SMI will not respond truthfully to the tool no matter what is included, resulting in false negatives. Whilst these challenges are well known [25] and important to highlight, none of the mental health consumers in this study reported being unwilling to use the tool, which is in line with the feedback previously highlighted regarding surgical patients with SMI wanting to have their mental health acknowledged [9]. The brevity and accuracy of the K10 has also been demonstrated as being a suitable tool for screening SMI in the general population [26]. Certainly, the planned research pilot will be critical for determining how or if the usefulness and thoroughness of the tool can be enhanced. Furthermore, helpful amendments were also suggested to improve the structure and language of the four additional questions, as well as the inclusion of a number of extra questions or response options, which will be incorporated into the amended tool for the pilot study. The results of the ‘Flesch Reading Ease’ and ‘Flesch-Kincaid grade level’ assessments also indicated simplification of the language to a lower grade level would be beneficial for patients.

The results of this study highlighted interesting variation in the scores provided by the different clinician groups that warrants further consideration. In assessing both the ‘usefulness’ and ‘thoroughness’ of the tools, the surgeons reported the highest and the psychiatrists the lowest consensus percentages for both tools with a statistically significant difference. Likewise, the ‘willingness of surgical patients to complete the tools preoperatively’ was reported with similar differences. Whilst limited inferences can be made from this data, these results possibly reflect the contrasting levels of expertise relating to mental health patients and experiences in screening them. Certainly, surgeons have previously reported a lack of confidence in managing surgical patients with mental illness [1] and may therefore have considered any tool that would assist in facilitating this process in a more positive light. Their experience with routinely screening for other medical comorbidities preoperatively may have also informed their impression that screening is a viable intervention that all patients would undertake. With a more storied experience, psychiatrists are likely to have been more knowledgeable about the range of challenges associated with screening patients about their mental health, and in particular for patients with SMI [25], and this may have been reflected in the lower scores they provided overall. It is evident that consideration of both perspectives is critical in being able to implement feasible interventions that help to bridge the gap for patients between the fields of surgery and psychiatry. Further examination of the details driving these points of view is needed.

Whilst this study makes an important contribution to the little researched area of mental health screening in the routine surgical setting, it does have limitations. The findings should be considered within the context of the participants involved and the single campus in which it was conducted. As both the clinician and consumer participants volunteered for the study, and we were unable to determine overall response rates, it may be possible they were biased or had particular experiences that compelled them to participate. The lack of demographic data collected also made this difficult to determine. Challenges in completing the online questionnaire were encountered by the mental health consumers with many opening the online link to the survey and selecting their participant grouping, but then not answering any questions, and thus not being included. Due to the anonymous nature of the study recruitment, it is not known why this was the case however it may reflect the online survey was not user friendly or straight forward to complete. Certainly it was a missed opportunity to seek their valuable feedback. It is evident that pilot testing the REDCap survey with a patient sample beforehand may have improved the completion rates and is an approach to be incorporated in future work. Furthermore, although important input was provided via the free-text item on the survey, there would be benefit for subsequent qualitative studies to examine in greater detail the areas that participants felt required attention including major benefits and drawbacks, along with the proposed administration process. Finally, due to the tools having previously been validated, this study was a high-level assessment of the tools as a whole in regard to their use within a routine surgical setting and did not involve participants considering individual items within the tools. There is an opportunity for future studies to undertake an assessment at a more granular level to further improve the tools for use in this unique setting. Similarly, whilst the methodological approach utilised to assess the tools was in line with other studies [21], there are limitations in using mean scores. It may be beneficial for further detailed assessments of the tools to apply different methodologies.

In conclusion, this study recommends the K10 mental health screening tool for preoperative use in the routine surgical setting. Important changes were highlighted as being required within the additional questions added to make the tool fit-for-purpose and focused on patients with SMI. Further development and testing is also needed to establish and strengthen the face validity of the K10 for this specific surgical cohort and purpose.

## Supplementary Information

**Additional file 1.** Additional questions added to the recommended mental health screening tools.

## Data Availability

The datasets used and/or analysed during the current study are available from the corresponding author on reasonable request.
