# Supplementary material for: Acceptability and face validity of two mental health screening tools for use in the routine surgical setting
Source: BMC Psychol. 2021 Oct 30;9:171. doi: 10.1186/s40359-021-00672-w (PMC8556895; doi:10.1186/s40359-021-00672-w)
Supplement: Supplementary file 1 — Additional file 1. Additional questions added to the recommended mental health screening tools. [file 40359_2021_672_MOESM1_ESM.docx]

**Supplementary Information 1:** Additional questions added to the recommended screening tools for preoperative use in the routine surgical setting

1. **Do you currently see or have you ever seen anyone for assistance with your mental wellbeing?**

| **□** | **□** | **□** | **□** |
| --- | --- | --- | --- |
| General Practitioner (GP) | Psychologist | Psychiatrist | Case Manager or Social Worker |
| **□** | **□** | **□** | **□** |
| NDIS Support Worker | Other clinician | Other: (*please describe)* ____________________________________ | No |

1. **Have you ever been told by a healthcare worker that you have one of following conditions?**

| **□** | **□** | **□** | **□** | **□** |
| --- | --- | --- | --- | --- |
| Anxiety | Depression | Bipolar Disorder | Schizophrenia | Alcohol or drug issues |
| **□** |  | **□** |  | **□** |
| Post-Traumatic Stress Disorder (PTSD) | | Other mental health condition  Please describe: ________________________________ | | No |

1. **Do you, or your carer / support person, have any serious concerns about your upcoming surgical procedure affecting your mental health and wellbeing? e.g. about your medication, your treatment, how you feel etc.**

| □ No I don’t  □ Yes I do → please describe: _________________________________________________  ___________________________________________________________________________  □ Yes my carer / support person does→ please describe: ___________________________  ___________________________________________________________________________ |
| --- |

1. **Would you, or your carer / support person, like to talk with someone about your mental health before your surgical procedure or receive information about mental health services available for you to use?**

□ No I don’t

□ Yes I do

□ Yes my carer / support person does
